# Induction of apoptosis in melanoma A375 cells by a chloroform fraction of *Centratherum anthelminticum* (L.) seeds involves NF-kappaB, p53 and Bcl-2-controlled mitochondrial signaling pathways

**DOI:** 10.1186/1472-6882-13-166

**Published:** 2013-07-10

**Authors:** Chung Yeng Looi, Bushra Moharram, Mohammadjavad Paydar, Yi Li Wong, Kok Hoong Leong, Khalit Mohamad, Aditya Arya, Won Fen Wong, Mohd Rais Mustafa

**Affiliations:** 1 Department of Pharmacology, Faculty of Medicine, University of Malaya, Kuala Lumpur, 50603, Malaysia; 2 Department of Pharmacy, Faculty of Medicine, University of Malaya, Kuala Lumpur, 50603, Malaysia; 3 Department of Medical Microbiology, Faculty of Medicine, University of Malaya, Kuala Lumpur, 50603, Malaysia

**Keywords:** *Centratherum anthelminticum*, Melanoma, Caspase cascade, Apoptosis, Bcl-2, p53, NF-κB

## Abstract

**Background:**

*Centratherum anthelminticum* (L.) Kuntze (scientific synonyms: *Vernonia anthelmintica; black cumin*) is one of the ingredients of an Ayurvedic preparation, called “Kayakalp”, commonly applied to treat skin disorders in India and Southeast Asia. Despite its well known anti-inflammatory property on skin diseases, the anti-cancer effect of *C. anthelminticum* seeds on skin cancer is less documented. The present study aims to investigate the anti-cancer effect of *Centratherum anthelminticum* (L.) seeds chloroform fraction (CACF) on human melanoma cells and to elucidate the molecular mechanism involved.

**Methods:**

A chloroform fraction was extracted from *C. anthelminticum* (CACF)*.* Bioactive compounds of the CACF were analyzed by liquid chromatography-tandem mass spectrometry (LC-MS/MS). Human melanoma cell line A375 was treated with CACF *in vitro*. Effects of CACF on growth inhibition, morphology, stress and survival of the cell were examined with MTT, high content screening (HSC) array scan and flow cytometry analyses. Involvement of intrinsic or extrinsic pathways in the CACF-induced A375 cell death mechanism was examined using a caspase luminescence assay. The results were further verified with different caspase inhibitors. In addition, Western blot analysis was performed to elucidate the changes in apoptosis-associated molecules. Finally, the effect of CACF on the NF-κB nuclear translocation ability was assayed.

**Results:**

The MTT assay showed that CACF dose-dependently inhibited cell growth of A375, while exerted less cytotoxic effect on normal primary epithelial melanocytes. We demonstrated that CACF induced cell growth inhibition through apoptosis, as evidenced by cell shrinkage, increased annexin V staining and formation of membrane blebs. CACF treatment also resulted in higher reactive oxygen species (ROS) production and lower Bcl-2 expression, leading to decrease mitochondrial membrane potential (MMP). Disruption of the MMP facilitated the release of mitochondrial cytochrome c, which activates caspase-9 and downstream caspase-3/7, resulting in DNA fragmentation and up-regulation of p53 in melanoma cells. Moreover, CACF prevented TNF-α-induced NF-κB nuclear translocation, which further committed A375 cells toward apoptosis.

**Conclusions:**

Together, our findings suggest CACF as a potential therapeutic agent against human melanoma malignancy.

## Background

Melanoma is a skin cancer that arises from the malignant transformation of melanocytes. Epidemiological studies showed that the incidence of melanoma is increasing at a rate faster than that of any other cancers worldwide [1-3]. Moreover, although early stage melanoma is confined to epidermis and is curable, metastasized melanoma has an unfavourable prognosis, where the overall survival for patients with metastatic melanoma ranges from 4.7 to 11 months, with a median survival of 8.5 months [4]. This poor prognosis is due to the lack of effective treatment options [5].

Melanoma is often characterized by resistance to cytoxic agents which contributes to the high morbidity and mortality rates in patients. Therefore it is important to look for new sources of anti-cancer agents that exert cytotoxicity activity against melanoma cells. Plant extracts have been used as complementary medicine for many years. Various phytoconstituents that possess multiple biological and synergistical effects in the plant extracts can function to treat different ailments or enhance the effect of drugs [6-9]. Certain natural products have been applied for cancer chemoprevention to inhibit or revert carcinogenesis and to suppress the malignancy of cancer [10].

*Centratherum anthelminticum* (L.) Kuntze seeds (scientific synonyms: *Vernonia anthelmintica*) is commonly known as black cumin and widely used as curry spice mixtures. Studies have shown various pharmacological properties exhibited in the seeds of *C. anthelminticum*, such as anti-viral, anti-filarial, anti-microbial, anti-fungal and anti-diabetic activities [11-15]. The black cumin seed is famous for its anti-inflammatory effect. It is used as an ingredient of an Ayurvedic preparation called “Kayakalp” to treat skin disorders and for body rejuvenation in India and Southeast Asia. Recently, a report showed that methanol extracts from *C. anthelminticum* promoted melanogenesis mainly by p38 MAPK activation, providing scientific explanation for its traditional use in skin disorders, such as leucoderma [16].

In comparison with other biological activities, the anti-cancer effect of CACF is relatively less documented. We recently reported that CACF inhibited the growth of breast cancer cell lines [17]; however, its effect and detailed mechanism on skin cancer is yet to be investigated. Hence, the aim of this study is to evaluate the therapeutic effect of CACF and the cellular mechanism underlying the anti-cancer effect on melanoma malignancy.

## Methods

### Plant materials

The seeds of *C. anthelminticum* plant were procured from the medicinal plant cultivation zone of Amritum Bio-Botanica Herbs Research Laboratory Pvt. Ltd, Betul Madhya Pradesh India. Voucher specimen (CA-9) was deposited in the Department of Pharmacology, University Malaya.

### Sample extraction

The seeds of *C. anthelminticum* (100 g) were pounded using grinder and extracted with hexane (3 × 250 ml) (Merck, Darmstadt, Germany) using soxhlet extractor. Thereafter, the residue obtained was further fractionated with chloroform (CHCl3) (3 × 250 ml) (Merck, Darmstadt, Germany) and finally with methanol (MeOH) (3 × 250 ml) (Merck, Darmstadt, Germany). The extract and crude fractions were collected, filtered and concentrated to dryness under reduced pressure in a rotary evaporator (<40°C). The hexane extract (CAHE) yielded 20.1 g, whereas, the defatted crude chloroform fraction (CACF) and the methanol fraction (CAMF) yielded 7.7 g and 11.6 g, respectively. Subsequent screening of the extract and fractions for their cytotoxicity, using the MTT assay, showed that the chloroform fraction (CACF) possesses a maximum of inhibitory effects against cancer cells. Therefore, CACF was chosen for further analysis.

### LC-MS/MS analysis

Liquid chromatography (LC) analysis was carried out using UFLC prominence series (Shimadzu Corp., Kyoto, Japan), equipped with a quaternary pump, a vacuum degasser, an autosampler, a column heater-cooler and PDA detector (diode array detector, DAD). Separation was accomplished using an XBridge C18 column (Waters, Ireland) (2.5 μm, 2.1 × 50 mm). About 1 mg of CACF was dissolved in 1 ml MeOH filtered through a 0.45 mm filter and subjected to high performance liquid chromatography (HPLC). Gradient elution was performed using a linear gradient solvent system consisting of solvent A (water with 0.1% formic acid) and solvent (B) (acetonitrile with 0.1% formic acid) as follows: 10–100% B over 7 min, followed by isocratic elution with 100% solvent (B) from 7–12.50 min, then returned to 10% from 13 min at a flow rate of 0.5 ml/min. The column temperature was maintained at 40°C and the injection volume was 10 μl. Separation of compounds was monitored with DAD at 254 and 190 nm and with a mass spectrometry detector.

Mass spectrometric analysis (ESI) was carried out on LCMS-8030 triple-quadrupole mass spectrometer (Shimadzu, Kyoto, Japan). Liquid chromatography–tandem mass spectrometry (LC-MS/MS) was set in the negative and positive ionization mode with spectra acquired over a mass range of 50–1000 m/z. The acquisition parameters were as following: interface voltage, 4.5 kV; interface temperature, 250°C; desolvation line temperature, 250°C; heat block temperature, 400°C; desolvation gas, nitrogen; desolvation gas flow rate, 3.0 l/min; drying gas, nitrogen; drying gas flow rate, 15 l/min; collision gas, argon; and collision gas pressure, 230 kPa.

### Cell culture

Human melanoma cell line (A375) was derived from the skin of a 54 year-old female patient with malignant melanoma [18]. This cell line was purchased from the American Type Culture Collection and cultured in DMEM media containing 10% Fetal Bovine Serum, 1% penicillin/streptomycin and maintained in a 37°C incubator with 5% CO_2_. Primary adult human dermal melanocytes (Cat. No.:2230) were purchased from Sciencell (Sciencell, San Diego, CA) and maintained in Melanocyte growth medium (Sciencell). All cells were maintained in an incubator at 37°C, 5% CO_2_.

### MTT assay

After 24 h of CACF treatment, 50 μl of MTT solution (2 mg/ml) was transferred to each well. Plates were incubated for 2 h at 37°C. Supernatant was discarded and DMSO was added to ensure total solubility of formazan crystals. Absorbance was recorded at 570 nm with Tecan Infinite^®^200 Pro microplate reader (Tecan, Männedorf, Switzerland).

### Real time cell growth assay

Cell proliferation was measured using xCELLigence Real Time Cellular Analysis (RTCA) system (Roche, Germany), as previously described [19]. Briefly, cells were seeded at density 1 × 10^4^ on a specialized 16-well plate with electrodes for 18 h before being treated with 100 μl of CACF at various concentrations and continuously monitored for up to 72 h. Cell index values were recorded every 5–10 min by RTCA analyzer and normalized to background reading.

### Flow cytometry analysis

CACF-treated cells were harvested and stained with FITC-annexin V and propidium iodide (PI) (BD Biosciences) in binding buffer for 15 min. Cells were immediately subjected to flow cytometry analyses using a FACS Canto II flow cytometer (BD Biosciences). For mitochondria membrane potential (MMP) measurement, CACF-treated cells were stained according to the BD™ MitoScreen kit protocol. Briefly, cells were stained with 0.5 ml of the JC-1 reagent for 15 min, washed and resuspended in 0.5 ml assay buffer prior to flow cytometric analysis.

### Bioluminescent assay for caspase-3/7, -8 and -9 activities

Caspase assay was performed in triplicates using the Caspase-Glo^®^-3/7, -8 and -9 assay kits (Promega, Madison, WI) on a white 96-well microplate. A total of 1 × 10^4^ cells were seeded per well and treated with 100 μl of CACF for 1, 3, 6, 12, 18, 24 and 30 h. Caspase-Glo reagent was then added and incubated at room temperature for 30 min. The caspase activities were measured using a Infinite^®^200 Pro microplate reader (Tecan). In the caspase inhibitor study, cells were treated for 1 h with inhibitors for pan-caspase (Z-VAD-FMK), caspase-3 (Z-DEVD-FMK), caspase-9 (Z-LEHD-FMK) or caspase-8 (Z-IETD-FMK), before CACF treatment.

### Transmission electron miscroscopy

Conventional electron microscopy was performed as described [20]. Cells were fixed with 2.5% glutaraldehyde in 0.1 M sodium phosphate buffer at pH 7.4 for 1 h. The specimens are post-fixed in buffer containing 1% osmium tetroxide (OsO_4_) and 1% potassium ferrocyanide, dehydrated in a series of graded ethanol solutions, and embedded in epoxy resin. Ultra-thin sections were collected and stained with uranyl acetate and lead citrate and observed under transmission electron microscope (TEM, Leo Libra 120, Germany).

### Multiple cytotoxicity assay

Cellomics Multiparameter Cytotoxicity 3 Kit was used as described previously [21]. Briefly, 24 h post CACF treatment, MMP dye and the cell permeability dye were added to live cells and incubated for 30 min at 37°C. Cells were fixed, permeabilized, blocked with 1× blocking buffer before probing with primary cytochrome c antibody and secondary DyLightTM 649 conjugated goat anti-mouse IgG for 1 h each. Plates were analyzed using the ArrayScan high content screening (HCS) system (Cellomics, PA, USA).

### ROS assay

A total amount of 1 × 10^4^ cells per well were seeded in 96-well plates overnight prior to incubation with CACF. At indicated time, 50 μl staining solution (DMEM containing 500 nM Hoechst 33342 and 2.5 μg/ml dihydroethidium (DHE) were added and incubated for 30 min at 37°C. Cells were fixed with 3.5% paraformaldeyde in PBS for 15 min, washed and analyzed using the HCS system (Cellomics).

### Western blot analysis

SDS-PAGE and Western blot analyses were performed as described with slight modifications [22]. At 24 h post treatment, cells were lysed in RIPA buffer, loaded onto 10% polyacrylamide gel and transferred to microporous polyvinylidene difluoride membrane (Milipore). Immunoblotting was performed with the anti-Bcl-2 (1:200), anti-p53 (1:200) (Cell Signaling Technology, Danvers, MA), and mouse anti-β-actin (1:500) (Sigma) antibodies. Membranes were detected using ECL Plus Chemiluminescence Reagent (Amersham, Chalfont, UK).

### Statistical analysis

Each experiment was performed at least two times. The data are presented as the mean values ± standard deviation (SD). Statistical analysis was performed with Student’s *t*-test, *P < 0.05 was considered statistically significant.

## Results

### LC-MS/MS analysis

**Figure 1:**
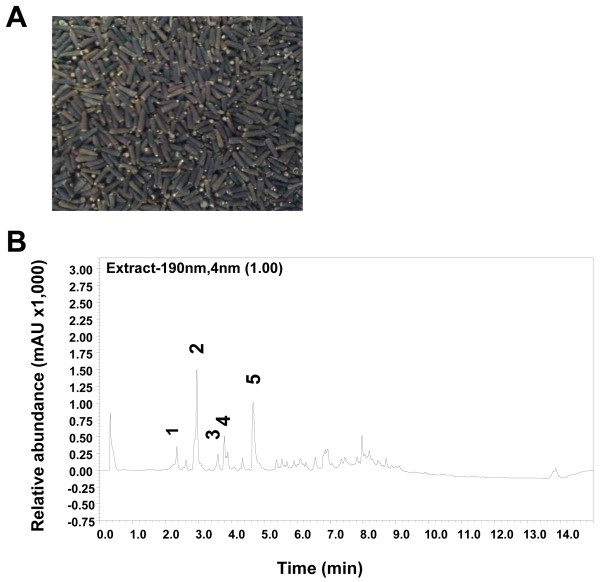
**Chemical contents of the chloroform fraction of *C. anthelminticum *seeds*. *(A) **Photo showing the seeds of *C. anthelminticum. ***(B) **LC chromatogram of CACF monitored at 190 nm. Peak 2, vernodaline; peak 3, vernudiflorid; peak 5, 12,13- dihydroxyoleic acid; peaks 1 and 4, unknown.

**Figure 2:**
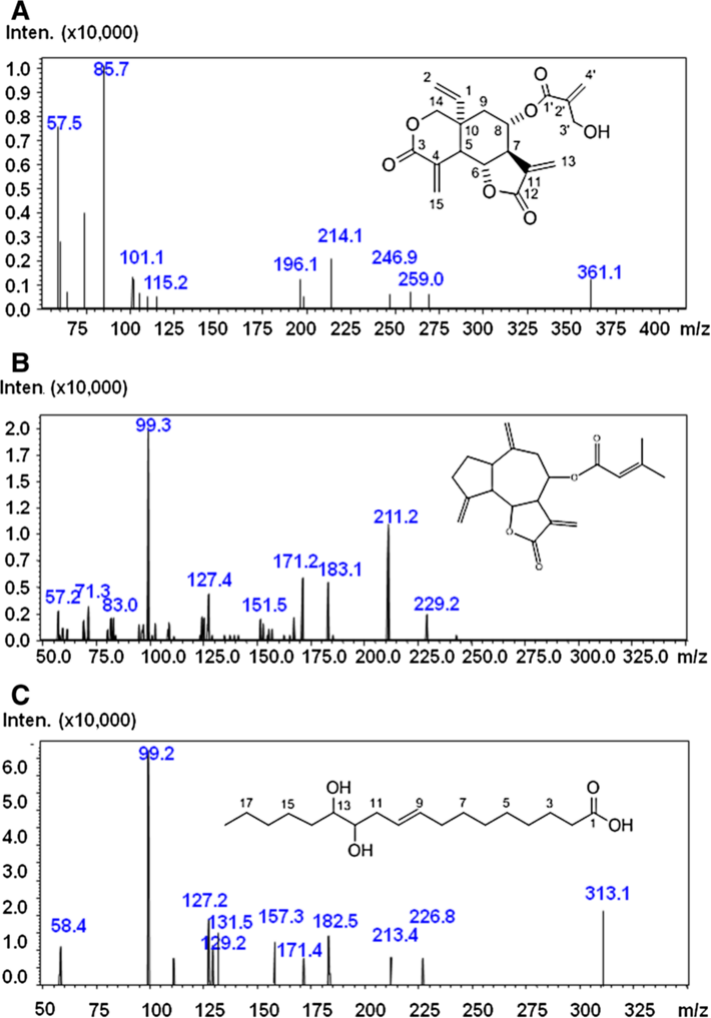
**MS/MS spectra and the corresponding chemical structure of the identified compounds. (A) **vernodaline, (**B**) vernuflorid, **(C) **12,13- dihydroxyoleic acid.

Compounds of CACF were successfully identified using LC-MS/MS. The UV chromatogram at 190 nm of CACF is shown in Figure 1. The compounds were identified by interpreting their mass spectra obtained via their MS, MS/MS, and UV spectra and comparing their data with those obtained in the literature. The identified compounds are as listed (Table 1) including retention times, molecular weight, protonated and deprotonated molecules ([M+H]+ and [M-H]-), MS/MS fragments, as well as their proposed identities. Five major peaks were observed in the chromatograms of CACF. Three compounds were identified by comparing their MS and MS/MS fragments data with those in literature. Peak 2 (Figure 2A), detected at 2.9 min, showed [M+H]+ at m/z 361 and was tentatively assigned to the sesquiterpene lactone, vernodaline, previously isolated from *Vernonia* species [19,23,24]. Its MS/MS spectrum gave fragmentation ions at m/z 57 and 85 which were attributable to the side chain of the compound. Peak 3 (Figure 2B), retained at 3.6 min, was identified as vernudiflorid. It showed molecular ion at m/z 329 [M-1]^-^ and fragmentation ions at m/z 229 and 99 corresponding to M- C_4_H_7_CO_2_H. These fragmentations were consistent with those reported for vernudiflorid, isolated from *Vernonia nudiflora*[25]. Peak 5 (Rt, 4.7 min; Figure 2C) showed [M-1]^-^ at m/z 313 and fragment ions at m/z 213, 182, 157 and 131. It was tentatively identified as 12,13- dihydroxyoleic acid. Ions at m/z 182 and 131 were obtained from the allylic cleavage indicated the double bond at C9 and C10. Fragment ions at m/z 213 and 157, derived from the alpha cleavage on the either side of the OH groups, h confirmed their positions at C12 and C13 [26,27]. This compound has been isolated previously from the seed of *C. anthelminticum* and *Ochrocarpus africanus*. The minor peaks 1 and 4 eluted at 2.3 and 3.8 min, respectively, were unidentified as their fragment ions did not match with previously reported compounds.

**Table 1:** Summary of compounds identified using MS, MS/MS, and UV spectra

**Peak #**	**Rt (min)**	**MW**	**[M+H]**^**+ **^**or [M-H]**^**-**^	**+/- Ions**	**MS/MS fragments**	**Tentative identification**	**References**
1	2.3	378	377	-	257, 257, 230, 187	unknown	
2	3.0	360	361	+	259, 247, 86, 58	vernodaline	[18,22,23]
3	3.6	330	329	-	229, 211, 99, 83	vernudiflorid	[24]
4	3.8	346	345	-	273, 209, 197,163, 84, 57	unknown	
5	4.7	314	313	-	213, 182, 157, 131,	12,13- dihydroxyoleic acid	[25,26]

Five peaks in the chromatograms of CACF (Figure 1B) were analyzed. Retention times (Rt), molecular weight (MW), protonated and deprotonated molecules ([M+H]+ and [M-H]-), +/- ions, MS/MS fragments, as well as their proposed identities are as listed. Among the five peaks, 2, 3 and 5 were identified as vernodaline, vernudiflori and 12,13- dihydroxyol, whereas the identities of minor peaks 1 and 4 remained unknown.

### CACF inhibits cell proliferation of melanoma cells

To evaluate the cytotoxic activity, CACF was tested with various doses on the human melanoma cell line A375 (highly metastatic, amelanotic) and normal human primary melanocytes. After 24 and 48 h, cell viability was analyzed using the end-point MTT assays. CACF exhibited greatest cytotoxicity on A375 cells with IC_50_ <10 μg/ml. In contrast, CACF was less toxic to normal primary melanocytes with IC_50_ >20 μg/ml, equivalent to a 2-fold higher selectivity compared to doxorubicin, the standard drug (Table 2).

**Table 2:** **IC**_**50 **_**of CACF or doxorubicin in melanoma A375 cells and normal primary melanocytes determined by MTT assays after 24 and 48 h treatment**

	**CACF**		**Doxorubicin**	
	**IC**_**50 **_**(mean ± SD) μg/mL**		**IC**_**50 **_**(mean ± SD) μg/mL**	
	**24 h**	**48 h**	**24 h**	**48 h**
**A375**	9.3 ± 2.5	8.5 ± 1.2	7.6 ± 0.8	6.2 ± 1.5
**Primary melanocytes**	24.3 ± 4.6	21.5 ± 3.1	8.5 ± 2.5	7.2 ± 2.2

**Figure 3:**
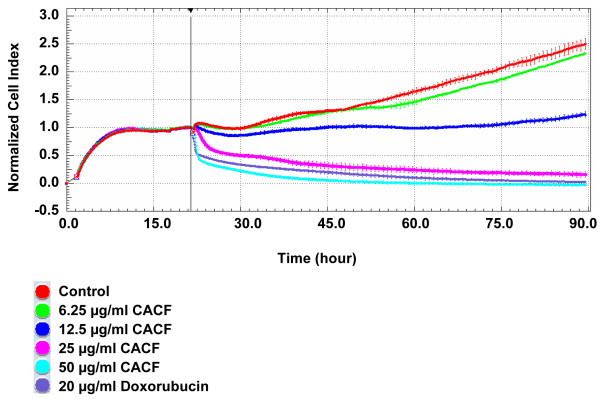
**Real time cell growth of CACF-treated melanoma cells. **A375 cells were seeded for 18 h before addition of CACF or doxorubicin (standard drug) at indicated concentrations. Cell growth was monitored by RTCA for up to 90 hours.

Next, we monitored the pattern of real time A375 cell growth after CACF treatment for 3 consecutive days using RTCA. In control wells, we observed an exponential increase of cell growth, as reflected by an increase normalized cell index (nCI) values. A375 cells treated with the standard drug, doxorubicin (20 μg/ml) demonstrated complete cell growth inhibition (Figure 3). A375 cells demonstrated a dose-dependent attenuation of cell proliferation, when treated with increasing concentrations of CACF (Figure 3). A375 proliferated at a slower rate at 6.25 μg/ml, whereas proliferation remained static when treated with 12.5 μg/ml CACF. A sudden decrease in nCI values was detected about 1–2 h after treatment with a high concentration (50 μg/ml) of CACF, indicating acute toxicity at high dosages. Altogether, both MTT and RTCA results suggest that CACF inhibited cell proliferation of A375 melanoma cells in dose- and time-dependent manner.

### CACF induces apoptosis in melanoma cells

**Figure 4:**
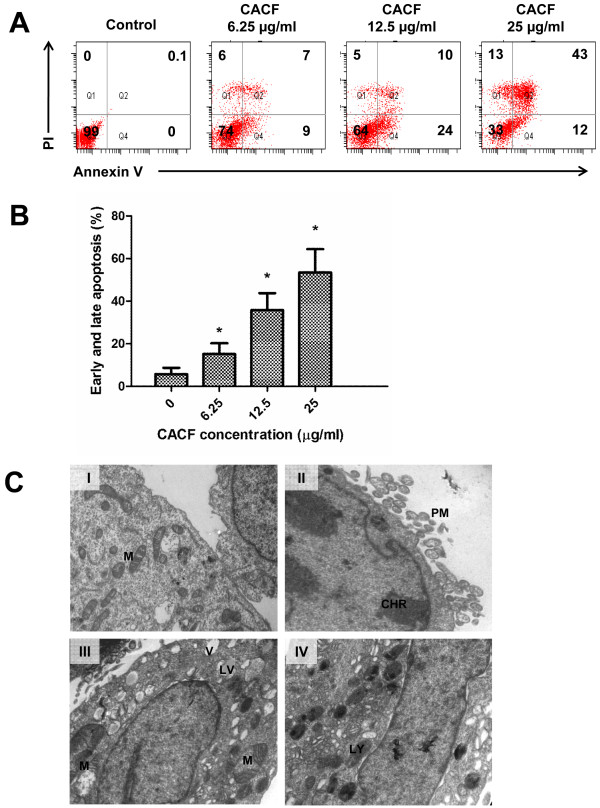
**Cell apoptosis upon CACF treatment. (A) **A375 cells were treated with DMSO or with various concentrations of CACF for 24 h. Cells were then stained with FITC-Annexin V and PI to analyze early apoptotic (Annexin V+ PI-) and late apoptotic/dead (Annexin V+ PI+) cell fractions through flow cytometry. **(B)** Histogram shows the percentages of apoptotic cells for each treated sample at 24 h after CACF treatment. Data were mean ± SD from 2 independent experiments (*P<0.05). **(C) **Transmission electron microscopy images of A375 cells untreated or treated with 12.5 μg/ml of CACF for 12 h. **(C-I) ** Representative image of untreated control cells. Intact cell membrane and abundant mitochondria were seen in the cytoplasm. **(C-II-IV) **Representative images of CACF (12.5 μg/mL)-treated cells. **(C-II) **Plasma membrane (PM) blebing and nuclear chromatin (CHR) condensation. **(C-III) **Increased numbers of clear vacuoles (V) with some lipid-filled vacuoles (LV). Reduced mitochondria copy number and disrupted cristae in the abnormally swollen mitochondria. **(C-IV) **Increased numbers of lysosome (LY) organelles.

To determine whether CACF-induced cell growth inhibition was due to apoptotic activity, we stained control or CACF-treated A375 cells with FITC-conjugated annexin V and PI. The exposure of phosphatidylinositol of the plasma membrane indicates early apoptosis and can be stained by annexin V. As shown in Figure 4A and B, significant increase of early (annexin V+, PI-) and late (annexin V+, PI+) apoptotic cells were detected in A375 following CACF treatment.

We further examined cell morphology by using transmission electron microscopy. Micrographs demonstrated that untreated control cells presented healthy morphology, including intact plasma membrane, normal nucleus and abundant numbers of mitochondria (Figure 4C-I). Upon CACF treatment, nuclear membrane condensation, disrupted cell structure and severe plasma membrane blebling were observed (Figure 4C-II), suggesting occurence of apoptosis [28]. Other drastic morphological changes included presence of many vacuoles with unknown content, probably of lipid origin (Figure 4C-III). Increased lysosome organelles were also presented in most of the CACF-treated cells. In the same line, the number of mitochondria were significantly reduced (Figure 4C-IV). Note that the mitochondria became swollen with disorganized cristae (Figure 4C-III). These collective data indicated that CACF induced apoptotic cell death in melanoma A375 cells.

### CACF induces high level of ROS

**Figure 5:**
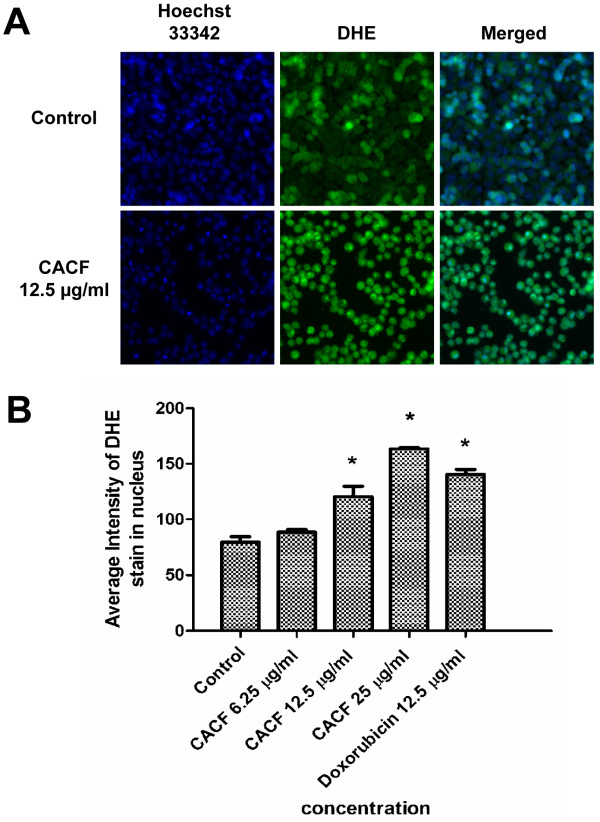
**ROS generation in CACF-treated cells. (A) **A375 cells were treated with DMSO or with 12.5 μg/ml concentration of CACF for 8 h, stained with DHE dye and visualized under HSC array scan reader. **(B) **Average fluorescence intensities of DHE dye in A375 cells treated with CACF or doxorubicin. Data were mean ± SD of fluorescence intensity readings measured from different photos taken (*P<0.05).

ROS is produced when a cell undergoes chemical or environmental stress, which can lead to modification of cytoskeletal structure and cell apoptosis. Next, we examined the ROS level in CACF-treated A375 cells by staining with DHE dye and viewed under HCS system. ROS production level was low in DMSO-treated control cells. However, CACF treatment strongly induced ROS production in A375 cells after 8 h (Figure 5).

### CACF treatment reduces MMP

**Figure 6:**
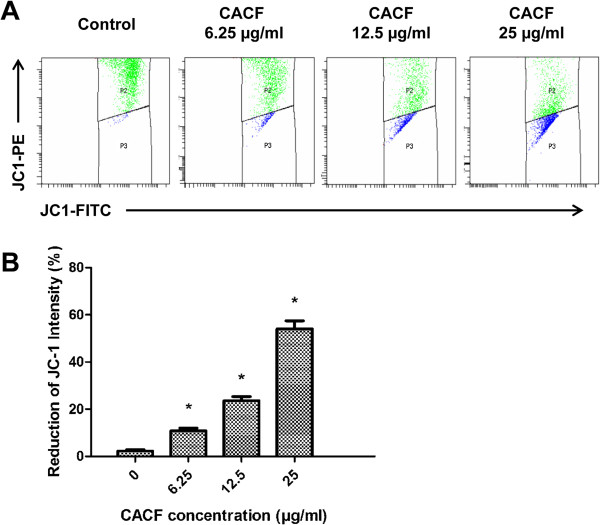
**JC-1 intensity in CACF-treated cells. (A) **A375 cells were treated with indicated concentrations of CACF for 12 h. Cells were then collected, stained and analyzed with the flow cytometer. **(B) **Bar chart shows the mean fluorescence intensity of JC-1-PE. Dose-dependent-reduction of JC-1-PE by CACF treatment was observed. Data were mean ± SD of fluorescence intensity readings from two independent experiments (*P<0.05).

Accumulating oxidative damage by ROS can affect the function and efficiency of mitochondria. To monitor the integrity of MMP, a membrane-permeable lipophilic cationic fluorescent probe, JC-1, staining was used (Figure 6A). In healthy polarized mitochondria, JC-1 molecules accumulate in mitochondria as aggregate, thus rendering strong red fluorescence emission. However, in apoptotic cells, JC-1 molecules stay as monomers in the cytoplasm, which can be reflected by weak red fluorescence emission [24]. Untreated A375 cells showed high intensity of JC-1-PE (upper quadrant). After 12 h CACF treatment, we observed a dose-dependent reduction of JC-1-PE intensity (lower quadrant) in melanoma cell indicating impaired MMP (Figure 6B).

### CACF treatment increases nuclear condensation, plasma membrane permeability and cytochrome c release

**Figure 7:**
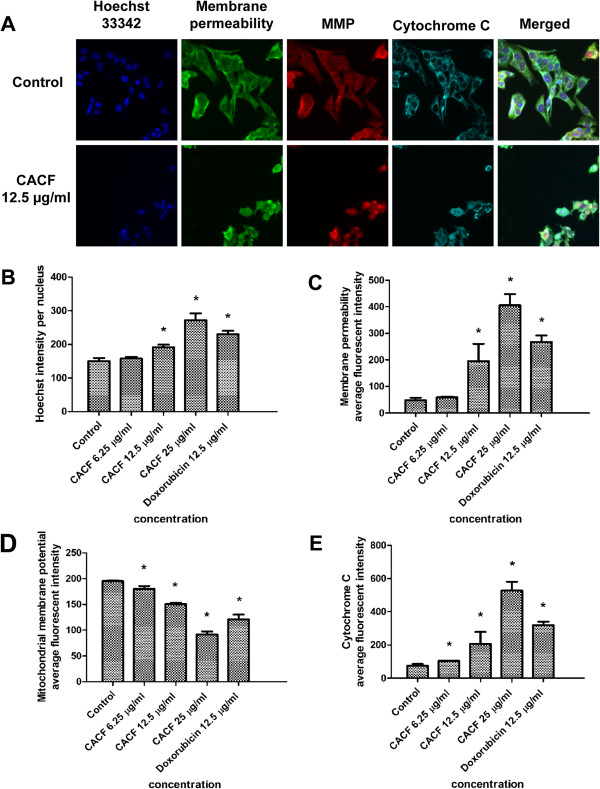
**Effect of CACF on MMP, permeability and cytochrome c release. (A)** Representative images of A375 cells treated with medium alone and 12.5 μg/ml of CACF, and stained with Hoechst for nuclear, cell permeability dye, MMP and cytochrome c. The images from each row were obtained from the same field of each sample (20×). **(B-E)** Average fluorescence intensities of Hoechst 33342, cell permeability dye, MMP and cytochrome c in A375 cells treated with CACF or standard drug doxorubicin. Data were mean ± SD of fluorescence intensity readings measured from different photos taken (*P<0.05).

Next, we utilised the multiparameter cytotoxicity kit 3 from Cellomics to examine the effect of CACF on other subcellular structures. For this purpose, we stained A375 cells with Hoechst 33342, a membrane permeability dye, MMP and cytochrome c antibody. The stained samples were visualized with the HSC system. In control cells, cytochrome c distributed evenly and colocalized with MMP. However, in CACF-treated cells, cytochrome c stained strongly in the cytosol, indicating CACF-induced cytochrome c release from the mitochondria (Figure 7A). In addition, we observed a dose-dependent increase of nuclear condensation, increased membrane permeability and decreased MMP stain in CACF-treated melanoma cells (Figure 7A-E).

### CACF induces caspase-9, -3/7 activity

**Figure 8:**
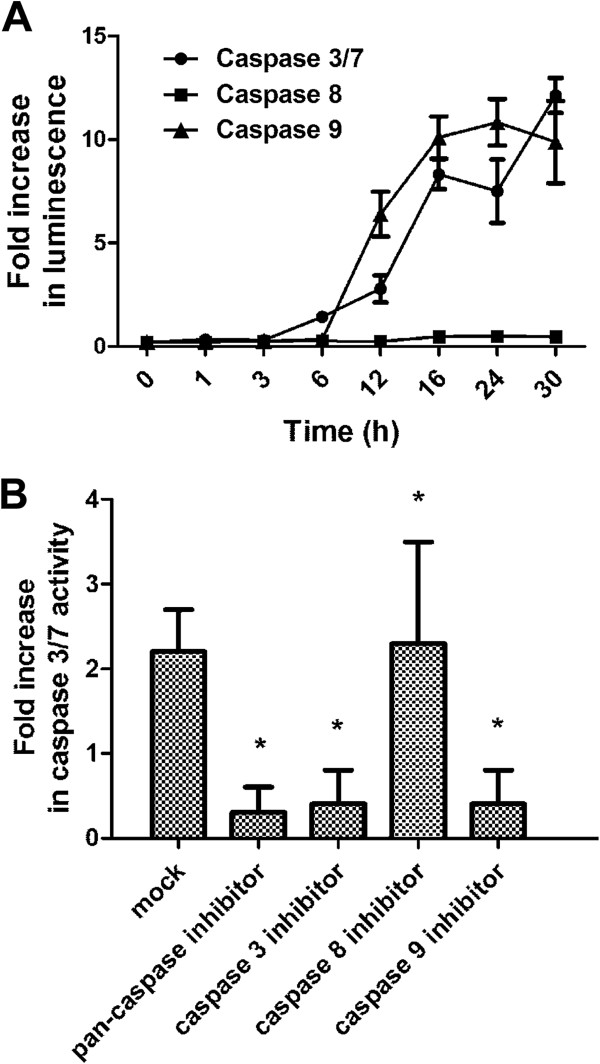
**Caspase activities in CACF-treated cells. (A) **Caspase-3/7, -8 and -9 activities in the CACF (12.5 μg/mL)-treated A375 cells were measured in luminescence against various time intervals. Activity of caspase-9 was increased followed by caspase-3/7 activities after CACF treatment. **(B) **A375 cells were pretreated with inhibitors for pan-caspase (Z-VAD-FMK), caspase-3 (Z-DEVD-FMK), caspase-9 (Z-LEHD-FMK) or caspase-8 (Z-IETD-FMK) before adding CACF. Caspase-3/7 activities in CACF-treated cells were then determined by luminescence assays. Data were mean ± SD of fluorescence intensity readings measured from different photos taken (*P<0.05).

Apoptosis is a complex phenomenon that mobilizes a number of molecules and is classified into caspase-dependent or caspase-independent mechanisms. To examine the molecular mechanism underlying the apoptosis process in CACF-treated A375 cells, we incubated cells with aminoluciferin-labeled caspase substrate, and caspase activities were determined by measuring luminescence intensities. After CACF administration, the activities of caspase-3/7, -8 and -9 were monitored for a period of 30 h. We observed a gradual increase of caspase-9 and 3/7 activities after 3 h treatment in A375 cells (Figure 8A). In contrast, there were no significant changes in caspase-8 activity throughout 30 h.

To further confirm this, we treated melanoma cells with specific caspase inhibitors. Pre-treatment with the pan-caspase (Z-VAD-FMK), caspase-3 (Z-DEVD-FMK) or caspase-9 (Z-LEHD-FMK) inhibitors, significantly impaired caspase-3/7 activity (Figure 8B). However, introduction of the caspase-8 inhibitor (Z-IETD-FMK) showed no significant inhibitory effect, suggesting that CACF-induced apoptosis occurred through the intrinsic, but not the extrinsic caspase pathway.

### CACF downregulates Bcl-2, upregulates p53 expression levels and inhibits TNF-α–induced NF-κB nuclear translocation

**Figure 9:**
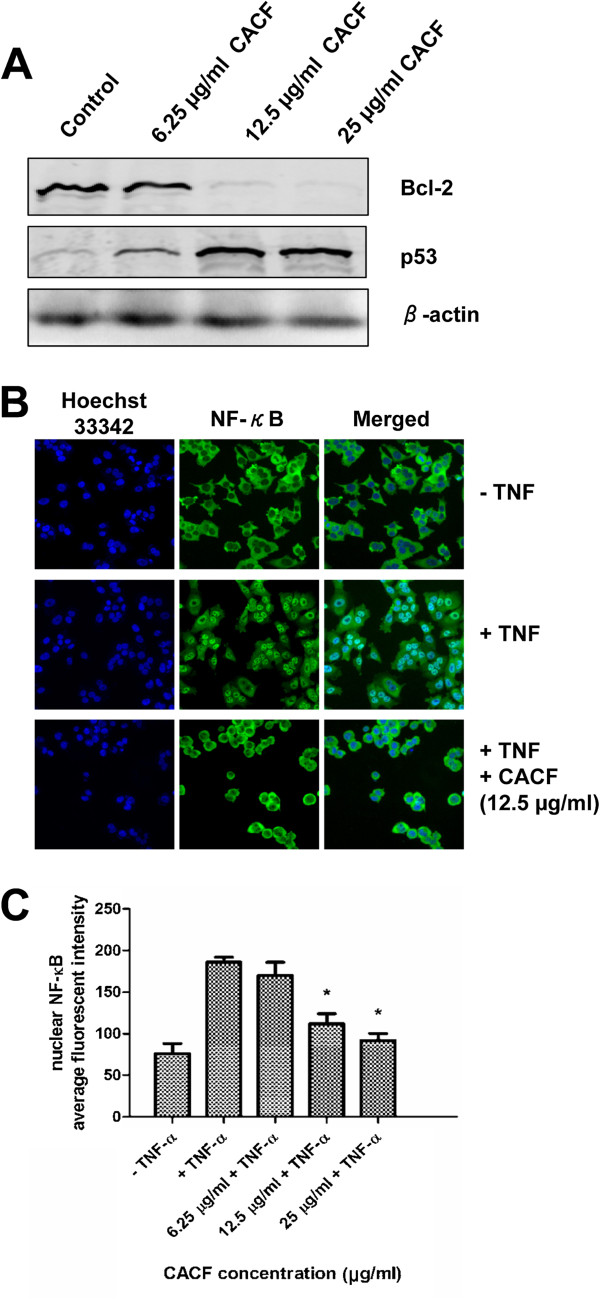
**Bcl-2, p53 expression and TNF-α-induced NF-κB translocation in CACF-treated cells. (A) **A375 cells were treated with various concentrations of CACF for 24 h. Cells were harvested and lysed for Western blot analysis. Membranes were probed with anti-Bcl-2, anti-p53 or anti-β-actin antibodies. Data were representative of two independent experiments. **(B) **A375 cells were pretreated with various concentrations of CACF prior to stimulation with 10 ng/ml TNF-α for 30 min. Cells were fixed, stained for NF-κB and visualized using HSC. **(C) **Bar chart showing the average fluorescence intensities of nuclear NF-κB in control/treated A375 cells in the absence/presence of TNF-α.

The anti-apoptotic molecule Bcl-2 is a key regulator of cell death and cell proliferation. Loss of Bcl-2 may lead to apoptosis activity, while enforced Bcl-2 expression in apoptotic cells inhibits cell death. To investigate the mechanism underlying CACF-induced cell death, we performed Western blotting to examine the expression level of Bcl-2 in human melanoma cells with/without CACF treatment for 24 h. Interestingly, we found that CACF dose-dependently downregulated Bcl-2 expression in A375 cells (Figure 9A).

One of the molecules that negatively regulates Bcl-2 is the p53 tumor suppressor protein. The p53 molecule is also frequently upregulated in response to DNA damage to exert cell cylce arrest [29]. Owing to this, we predicted that p53 expression could be affected upon CACF treatment. As shown in Figure 9A, we demonstrated that CACF markedly increased the expression of p53 protein in melanoma cells.

**Figure 10:**
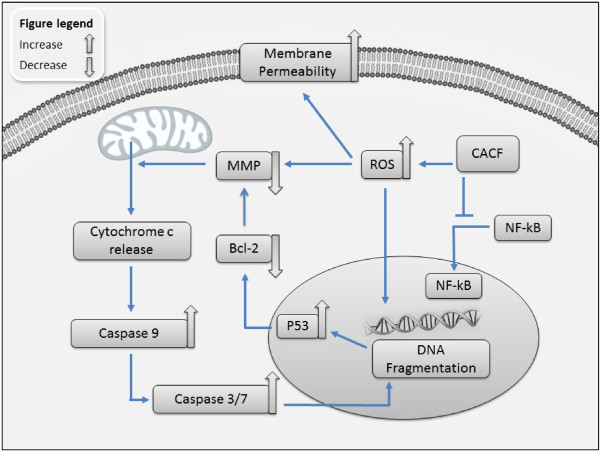
**Schematic model of cell death mechanism in CACF-induced responses in melanoma A375 cells. **CACF induces intracellular ROS production due to elevated oxidative stress. This initiates various intracellular changes including increased membrane permeability and reduced MMP which subsequently causes cytochrome c release from the mitochondria. Cytoplasmic cytochrome c activates caspase pathway molecules, including caspase-9 and caspase-3/7. Activation of caspase molecules subsequently leads to nuclear fragmentation. CACF treatment also triggers p53 expression that may initiate apoptosis activity through reduction of anti-apoptotic Bcl-2 level. On the other hand, CACF prevented NF-κB nuclear translocation, which could induce Bcl-2 transactivation and maintain cell survival in A375 melanoma cells.

The NF-κB pathway confers resistance to apoptosis through induction of Bcl-2 expression [30]. We hypothesized that reduced Bcl-2 expression may be due to inhibition of the NF-κB pathway. To address this, we treated A375 cells with CACF prior to addition of TNF-α. Cells were fixed, stained and visualized as described in the Materials and Methods. In the absence of TNF-α (-TNF), NF-κB remained in the cytoplasm (Figure 9B). TNF-α stimulation (+TNF) led to translocation of NF-κB from cytoplasm to the nucleus (Figure 9B). Interestingly, NF-κB nuclear translocation induced by TNF-α was significantly inhibited at 12.5 μg/ml and 25 μg/ml of CACF (Figure 9B and C). Mechanism of NF-κB nuclear translocation involves cascade reactions including the phosphorylation activity by IκB kinase (IKK), ubiquitin-dependent degradation of IκB and dissociation of NF-κB from the IκB complex. Therefore, CACF may interrupt these activities downstream of TNF signaling pathway which prevent NF-κB nuclear migration. Together, these data suggest a potential interplay between NF-κB and apoptosis pathway molecules during CACF treatment (Figure 10).

## Discussion

Malignant melanoma remains a great challenge due to its significant resistance to chemotherapeutic treatments, which is mostly caused by an intrinsic resistance of the neoplastic melanocytes to undergo apoptosis [31]. Although both active and passive immunotherapy have been pursued vigorously over the past few decades, no melanoma vaccine has proven effective, and only interleukin 2 therapy has led to durable remission in only 5–8% of patients treated [32]. In this context, plant-derived compounds play an important role in the development of new anti-cancer agents against human melanoma.

In this study, the fraction of CACF was analyzed by LCMS-IT-TOF, in order to correlate the activity of CACF with its components. Vernodalin, a sesquiterpene lactone, was the predominant compound in the CACF fraction. In 1969, an investigation by Kupchan et al. [23] revealed tumor inhibitory activity of vernodalin against cells derived from human carcinoma of the nasopharynx (KB). Similar findings were reported by Kasim et al. in 2011, where vernodalin indicated significant cytotoxic activity against the melanoma (Sk-mel 28) and ovarian cancer (CAOV-3) cell lines [33]. Recently, our group showed that vernodalin possessed strong cytotoxicity against the breast cancer cell line (MCF7) via the caspase pathway [19]. Thus, vernodalin could be the cytotoxic compound responsible for the CACF-induced cell growth inhibition in melanoma cells, although further research is needed to verify this.

Our collective data suggest that A375 melanoma cells undergo apoptotic cell death after treatment with CACF. During early apoptosis, the mitochondrial transmembrane depolarizes, followed by cytochrome c leakage which triggers caspase cascade activation [28]. Caspases belong to family of cysteine proteases that are divided into executioner (caspase-3 or -7) and initiator (caspase-8 or -9) caspases. Initiator caspase-8 is known to be activated through the extrinsic pathway, whereas caspase-9 is activated in the intrinsic event of mitochondrial cytochrome c leakage. Both of these initiator caspases lead to downstream activation of executioner caspase-3 or -7, which triggers cell apoptosis [34]. Incubation with CACF resulted in a time-dependent activation of caspase-9, whereas caspase-8 was rather unaffected throughout the period of study. In addition, treatments with Z-VAD-FMK, Z-DEVD-FMK or Z-LEHD-FMK caspase inhibitors significantly impaired caspase-3/7 activities. Thus, our results clearly implicate the involvement of the mitochondria-dependent intrinsic apoptotic pathway in CACF-induced melanoma cell death.

ROS are highly reactive molecules that can oxidize lipids, proteins, and DNA. Mitochondria provide metabolic energy through oxidative phosphorylation and generate ROS as normal side product. Natural compounds (e.g. curcumin) or anti-cancer drug (e.g. doxorubicin) can upregulate intracellular ROS level and signal cells to differentiate or undergo apoptosis. Excessive production of ROS by CACF could destroy MMP (marked by low JC-1 staining), which eventually leads to release of proapoptotic transducing molecules such as cytochrome c. In addition, ROS could also destroy plasma membrane and DNA, resulting in higher membrane permeability staining and DNA fragmentation, as observed in this study [35].

In this study, we found that anti-cancer activity of CACF may be attributed to Bcl-2 downregulation in melanoma cells. It is well established that Bcl-2 generally acts as a potent anti-apoptosis gene by controlling several key steps in apoptosis signaling, including formation of ion channels in biological membranes that influence permeability of intracellular membranes and cytochrome c release from mitochondria. Therapeutic agents that target Ras/Raf signaling often face a resistance problem, whereas a new strategy that target the survival Bcl-2 family proteins (Mcl-1 and A1) has been successful and further enhanced with chemotherapy [36]. Thus, Bcl-2 downregulation by CACF is an effective way in sensitizing human melanoma cells to apoptosis.

In addition to the anti-apoptotic Bcl-2 molecule, we showed that p53 expression is up-regulated in CACF-treated melanoma cells. Increased p53 may be triggered by nuclear fragmentation to repair DNA damage. Loss of p53 expression in mice and in human melanocytes has been shown to increase the proliferation and *in vivo* tumourigenicity, concordant with the role of p53 as a tumour suppressor [37,38]. Interestingly, we also showed that NF-κB translocation into the nucleus is inhibited by CACF, which could subsequently affect the expression levels of Bcl-2 and p53, because both of these molecules are downsream transcription targets of the NF-κB pathway [30,39,40].

In this study, we have demonstrated anti-cancer activity of CACF on human malignant melanoma A375 cell line. The limitation of the current study can be further expanded in future investigations by evaluating CACF therapeutic effects on other melanoma cell lines or *in vivo* xenograft model. These findings will help to explore the applicability of the plant extract and its constituents on skin cancers.

## Conclusion

This report shows that CACF has profound cytotoxic activity against human melanoma cells, but not normal melanocytes, by inducing apoptosis through modulation of anti- and pro-apoptotic signaling pathways (Figure 10). CACF induces intracellular ROS generation, which causes DNA damage, increased membrane permeability and activation of a mitochondria-dependent caspase cascade. These effects induce upregulation of p53 and downregulation of Bcl-2, which commit cells to apoptosis. In addition, CACF also inhibits pro-survival signaling by preventing NF-κB nuclear translocation. Together, our findings further support the development of CACF as an alternative therapeutic agent against melanoma malignancy.

## Abbreviations

CACF: *Centratherum anthelminticum* seeds chloroform fraction; CAHE: *Centratherum anthelminticum* seeds hexane extract; CAMF: *Centratherum anthelminticum* seeds methanol fraction; DAD: Diode array detector; DHE: Dihydroethidium; HPLC: High performance liquid chromatography; LC-MS/MS: Liquid chromatography–tandem mass spectrometry; MMP: Mitochondria membrane potential; MeOH: Methanol; nCI: Normalized cell index; PI: Propidium iodide; ROS: Reactive oxygen species; RTCA: Real-time cellular analysis.

## Competing interests

The authors declare that they have no competing interests.

## Authors’ contributions

CYL, AA and MRM conceived the study. CYL, MP and YLW designed, performed experiments and analyzed the data. BM conducted LC-MS/MS analysis. WFW conducted annexin staining for flow cytometry analysis. KHL and KM conducted caspase assay and JC-1 staining. CYL and BM wrote the manuscript. All authors read and approved the final manuscript.

## Pre-publication history

The pre-publication history for this paper can be accessed here:

http://www.biomedcentral.com/1472-6882/13/166/prepub
